# Development of an investigational medicinal stem cell gene therapy product to attempt curing human immunodeficiency virus infection

**DOI:** 10.1038/s41598-026-64038-1

**Published:** 2026-08-04

**Authors:** Laura Weigand, Stefanie Herkt, Maike Voges, Niklas Beschorner, Juliane Friedrich, David Rieger, Darja Karpova, Britta Weseloh, Laura Vogel, Jan Chemnitz, Halvard Bonig, Eliza Wiercinska

**Affiliations:** 1 Institute for Transfusion Medicine and Immunohematology, and German Red Cross Blood Service BaWüHe, Goethe University, Frankfurt, Germany; 2 PROVIREX Genome Editing Therapies GmbH, Hamburg, Germany; 3 Faculty of Medicine, Institute for Transfusion Medicine and Immunohematology, Goethe University, Sandhofstr. 1, 60528 Frankfurt a.M., Germany

**Keywords:** HIV, Brec1, Stem cell gene therapy, HSCE process, CliniMACS Prodigy, Biotechnology, Diseases, Immunology, Microbiology, Stem cells

## Abstract

For most people living with Human immunodeficiency virus (HIV), virus control and sufficient immune function can be achieved with polypharmacotherapy. A minority is intolerant to thereto, and drug-resistant mutants are emerging. Additionally, people living with HIV (PLWH) have significantly higher risk of cardiovascular morbidity and malignant diseases. HIV is in principle immunogenic. If T-cell depletion could be avoided immunological HIV eradication should be achievable. The “Berlin patient” in whom allogeneic stem cell transplantation from a CCR5Δ32/Δ32 donor induced long-term remission without antiviral therapy raised the hope that stem cell gene therapy with CCR5-deleted autologous stem cells could provide a cure, but the strategy did not live up to expectations. We propose to test an alternative strategy, namely expression of a designer recombinase which specifically excises HIV provirus from the genome under a Tat-inducible promoter so that expression is restricted to infected cells. A cGMP-compliant manufacturing protocol and quality control strategy for this investigational medicinal product was developed, validated, and a manufacturing authorization obtained. A First-in-Human-clinical trial, testing engraftment, repopulation of peripheral specific immunity, and ability to control HIV infection without antiviral medication is in preparation. Protocols can be adapted to other stem cell gene therapies by transferring alternative lentviral cargo.

## Introduction

HIV provirus inserts into the genome of infected patients, establishing latent pools from where virus can re-activate when antiviral drugs are interrupted. The main pool of latent HIV are T helper cells; whether hematopoietic stem/progenitor cells can also harbor HIV has been discussed controversially^1^. The conventional therapeutic goal in HIV infection is effective viral suppression, which is typically capable of maintaining sufficient immune function and preventing development of AIDS, but does not constitute a cure. Side effects of the polypharmaceutical regimes (antiretroviral therapy, ART) are frequent and occasionally limiting. Moreover, resistant mutants are beginning to emerge^2,3^ and ~ 1% of people living with HIV do not respond to any of the existing ART classes and develop AIDS symptoms^4^.

Despite effective long‑term ART, a substantial proportion of people living with HIV (PLWH) continue to exhibit chronic systemic immune activation and associated immune dysregulation, which may contribute to cardiovascular disease and other inflammation‑driven comorbidities^5–7^. In addition, the risk of developing malignancies remains significantly elevated even under successful ART^8–10^. PLWH also continue to experience substantial stigma related to their infection^11,12^.

Taken together, these factors underscore the ongoing medical need for therapeutic strategies capable of achieving ART‑free HIV remission or, ideally, a definitive cure.

Potentially, HIV infection could be cured if cells harboring latent virus were eradicated, or latent virus could be removed. The cures for HIV reported so far were for patients undergoing allogeneic hematopoietic stem cell transplantation for curing blood cancers. The donors had a homozygous mutation of the HIV co-receptor CCR5 (CCR5Δ32), which leads to a resistance of HIV infection^13–17^. Stem cell transplantation from CCR5Δ32/Δ32 donors is potentially curative of HIV infection with CCR5-tropic strains, as evidenced by four carefully analyzed cases. A greater number of such treatments, however, was not successful: Some CCR5Δ32/Δ32 transplant recipients died from relapse of the underlying hematological malignancy, graft rejection, graft-versus-host disease, or infectious disease complications before long-term HIV remission could be assessed. Furthermore, rebound with CXCR4-tropic viruses has been reported in cases where durable HIV remission was not achieved. Gene editing of CCR5 in autologous T-cells failed to control HIV, likely due to a combination of insufficient gene editing efficiency and acquired functional deficits in long-term engraftment of the gene edited cells^18^. As reported last year, even a heterozygous CCR5Δ32 stem cell transplantation resulted in treatment-free HIV remission^19^. Given the low probability of finding an HLA-matched hematopoietic stem cell donor deficient for CCR5, despite the modest success rate of curing HIV even when it is achieved, several groups sought to cure HIV by inducing CCR5 deficiency with zinc-finger-nucleases (ZFN), transcription activator-like effector nucleases (TALEN), or CRISPR/Cas^20–24^. In addition, stem cell gene therapy could be another alternative approach to address HIV.

In order to more directly target the integrated proviral DNA we developed a HIV-specific recombinase by molecular evolution technique^25^. The site-specific recombinase (SSR) Brec1 specifically recognizes the 34 bp loxBTR sequence that is present in the LTR R-region of the vast majority of HIV-1 primary isolates and has been shown to excise the HIV provirus in vitro and in vivo. Brec1 was generated as a derivative of Cre recombinase by molecular substrate linked protein evolution^25^. In contrast to naturally occurring recombinases such as Cre and others, Brec1 recognizes an asymmetric recognition site with high specificity.

Brec1 excises proviral DNA with high specificity and nucleotide-level precision. This performance reflects a key conceptual advantage of recombinase-based mechanisms: they do not generate overt double-strand breaks nor rely on cellular DNA repair pathways^25,26^, in contrast to CRISPR/Cas9-mediated excision. Whereas CRISPR/Cas systems initiate cleavage simply upon protein binding to the DNA-guide RNA hybrid, Brec1-driven recombination proceeds through a coordinated, multistep spatio-temporal process governed by association-dissociation kinetics at two recognition sites in parallel.

Brec1-mediated recombination occurs between two loxBTR recognition sites. Each loxBTR site consists of two half-sites separated by a spacer region. In the initial step, the two half-sites are bound by two Brec1 molecules through sequence‑specific protein-DNA interactions, followed by dimerization of the bound Brec1 molecules. The same sequence of events must occur in parallel at the second loxBTR site. In the subsequent step, the two dimers assembled at the two loxBTR sites associate to form a tetrameric recombination complex, which is required for catalysis. This precise, highly coordinated mode of action enables Brec1 to reduce viral load below the detection limit both in vitro and in HIV‑infected humanized mice^25^.

Assessment of recombination at potential off‑target sites described by Karpinski et al.^25^ and Bessen et al.^27^ requires consideration of four independent criteria. First, the half‑sites of the putative off‑target locus must exhibit sufficient sequence homology to the loxBTR recognition sequence to permit Brec1 binding. Second, the spacer regions of the two putative loxBTR sites must be nearly identical, as complementary hybridization of these spacers is essential for recombination by the Brec1 tetramer^28,29^. Third, the genomic distance between the two candidate sites must fall within a range that still allows tetramer formation. Recombination efficiency of site‑specific recombinases such as Brec1 declines approximately exponentially with increasing genetic distance, decreasing from ~ 11% at 2 cM to < 10⁻⁶ at 60 cM^30^. The fourth requirement concerns the probability that all four Brec1‑binding events occur simultaneously to enable tetramer assembly, which depends on association-dissociation kinetics and the local concentration of Brec1. Taken together, these constraints indicate that non‑specific recombination of Brec1 at the potential off‑target sites proposed by Karpinski et al.^25^ and Bessen et al.^27^ is exceedingly unlikely, as all four conditions would need to be met concurrently.

Thanks to a Tat-inducible promoter, Tat being the first HIV protein to emerge after HIV infection (or reactivation of latent reservoirs), this recombinase was shown to be induced specifically in HIV-infected cells, adding a second layer of biological (genetic) safety. T-cells harboring Broad-range recombinase 1 (Brec1)-Tat-inducible gene could thus be effectively cleared of HIV proviral DNA after infection, de facto leading to functional resistance to a productive HIV infection^25,31^.

Packaged into lentiviral vector particles, it could stably transduce primary human stem/progenitor cells and be passed on to all their progeny, where Brec1 could be activated by transfection with recombinant Tat, while the cells retained engraftment and multi-lineage differentiation potential in immunodeficient mice^25,32^.

We propose that transplantation of autologous Brec1 engineered hematopoietic stem/progenitor cells in Busulfan-conditioned recipients will establish Brec1 chimerism in all hematopoietic lineages. Subsequently, we predict that after discontinuation of ART the ensuing viremia will elicit anti-HIV immune responses in the HIV-resistant Brec1-modified T-cells emanating from the graft, in which case clearance of HIV infection would be achieved by the combination of HIV-resistance of engineered cells and adaptive immune responses. To test this hypothesis, a good manufacturing procedure (GMP) compliant manufacturing process and quality control strategy was established and formally validated, and a manufacturing authorization acc. § 13 of the German Medicines Law for manufacturing this investigational medicinal product (IMP) was obtained.

Pharmaceutical development and results of the validation runs are presented here. Besides documenting progress towards the potential cure of HIV, the data presented here especially for the quality control assays may be useful to other groups seeking to establish stem cell gene therapy production processes.

## Results

**Fig. 1:**
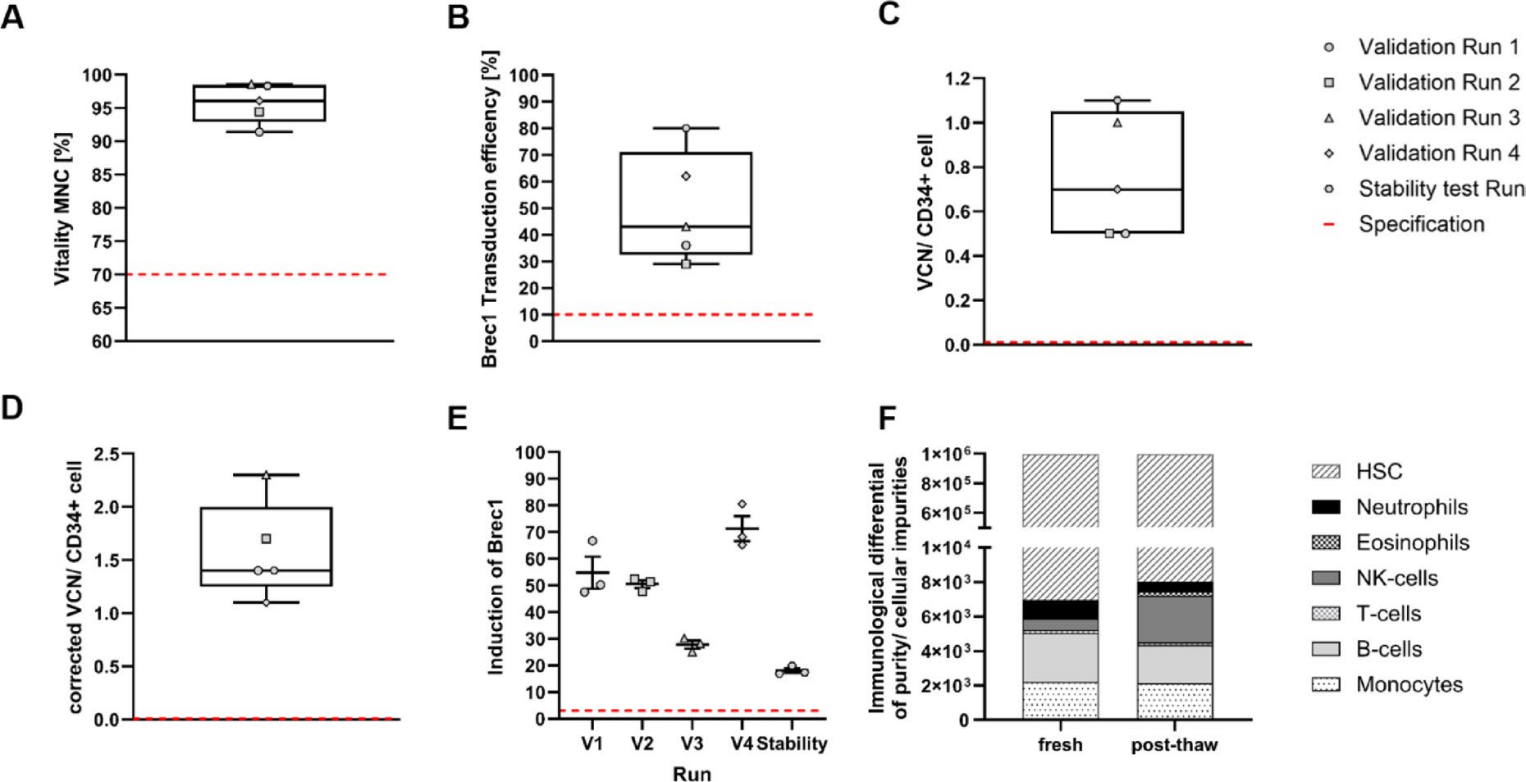
Quality control analysis on final drug product. **(A)** Vitality of the drug product was determined via flow cytometry post-thaw; all products fulfilled the specification of ≥ 70%, **(B)** Transduction efficiency was measured on harvested colonies via qPCR by comparing the fraction of the gDNA (RnaseP) control-positive PCR reactions with those positive for Brec1. The specification of > 10% for transduction efficiency was fulfilled by all products. **(C)** VCN was determined on harvested colony assays by assessing the relative number of Brec1 positive vs. gDNA (RPP30) control positive drops. The release criteria for VCN is > 0, which was exceeded by all products. **(D)** Corrected VCN was calculated by multiplying transduction efficiency and VCN (for information only). **(E)** Functionality of the ATMP was determined by testing the induction of Brec1 after stimulation with HIV-1 Tat. The release criterion of “inducibility”, met if Brec1 mRNA is increased over baseline by ≥ 3, was achieved in all cases. **(F)** Contaminating mature white blood cells were analyzed in drug substance (fresh) and post-thaw drug product (post-thaw) (mean from all manufacturing campaigns per million WBC; for information only).

All four validation runs and the stability run proceeded without any relevant deviations, and all products met all specifications (Table 1; Fig. 1). The starting cell doses were highly variable and did not quite reach full-size ATMP manufacturing runs for a typical adult, as excess cells from good mobilizing pseudonymized healthy donors donating for allogeneic recipients were used. After CD34 + cell enrichment, 58.9% (range: 53.6–64.4%) of CD34 + cells were recovered from the apheresis. After transduction on Prodigy, 81.6% (range: 56.1–99.0%) of the initially seeded cells could be harvested and frozen. After thawing, the mean viability was 95.8% (range: 91.4–98.6%) (Fig. 1A). Thus approximately 48.2% (range: 34.3–63.8%) of initially collected CD34 + cells were recovered after enrichment with CliniMACS Plus, transduction and cultivation from the CliniMACS Prodigy.

All products were negative for microbial growth and mycoplasma by NAT, as well as endotoxin was below the lower limit of detection of 0.8 IU/mL. Purity of CD34 + cells after enrichment exceeded 98.0% (range: 96.5–98.8%). Contaminating mature cells measured post thaw in the final drug product were very low, < 1%. They consisted of 2700 NK cells, 2200 B-cells, 2100 Monocytes, 900 Neutrophils, 200 Eosinophils and 100 T-cells per 10^6^ cells (Fig. 1F). Transduction efficiency was 50.0% (range: 29.0–80.0%) (Fig. 1B), vector copy number was 0.76 (range: 0.5–1.1) (Fig. 1C), corrected VCN was 1.58 (range: 1.1–2.3) (Fig. 1D) and clonogenicity was always given (all on post-thaw drug product). An at least 18-fold increase (median: 44.5; range: 18.1–71.3; specification: >3) of the Brec1 mRNA PCR signal was observed after electroporation with recombinant Tat protein (Fig. 1E).

The totality of quality control results and release criteria is given in Table 1 together with the targeted specification for each of the parameters.

**Fig. 2:**
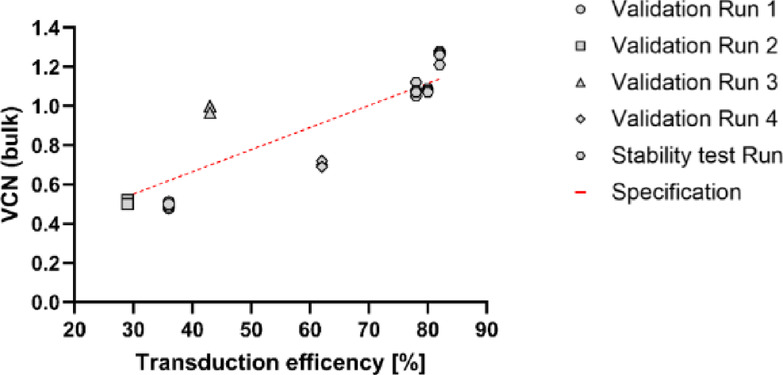
Correlation between transduction efficiency (TE) and vector copy number (VCN). TE and VCN measurements from the all five manufacturing runs and the values from later time points of the stability study are presented in a correlation analysis, indicating a significant correlation of the two measures as well as high precision of the analytical methods used for their determination. Each dot represents one measurement from a given manufacturing run.

Since a set of 96 picked clones for assessment of transduction efficiency represents a relatively small sample size, we ascertained how well the transduction efficiency measured in these 96 clones correlates with the vector copy number (VCN) determined from a bulk population comprising several tens of thousands of cells. As shown in Fig. 2, the two parameters exhibit a strong correlation, with an R² value of 0.7. Aliquots from the stability run were analyzed so far three times over several month as part of a stability study. These repeated measurements show excellent reproducibility (the three data series clustered around ~ 80% transduction efficiency in Fig. 2). Taken together, these results demonstrate that the validated methods provide precise, reliable and consistent measurement of both VCN and transduction efficiency.

**Table 1:** Pre-defined release criteria and specifications of five manufacturing runs.

	Test parameter		Specification	Validation Run 1		Validation Run 2		Validation Run 3		Validation Run 4		Stability test Run	
CD34 + Selection	**Microbial examination of cell-based preparations** (Ph. Eur. 2.6.27) after CD34 + selection		no growth	neg.		neg.		neg.		neg.		neg.	
	**Volume [mL]**		determined, declared	80 mL		80 mL		81 mL		82 mL		38 mL	
	**WBC total [×10** ^**6**^ **]**		determined, declared	307.6 × 10^6^		142.9 × 10^6^		1154.3 × 10^6^		959.2 × 10^6^		186 × 10^6^	
	**Vitality CD34+ [%]**		≥ 80%	99.0%		98.5%		99.6%		99.3%		98.8%	
	**Purity CD34+ [%]**		≥ 90%	97.9%		96.5%		99.1%		97.4%		96.6%	
	**CD34 + total [×10** ^**6**^ **]**		determined, declared	301.3 × 10^6^		138.0 × 10^6^		1143.4 × 10^6^		934.3 × 10^6^		179.8 × 10^6^	
Drug Substance	**Microbial examination of cell-based preparations** (Ph. Eur. 2.6.27) culture medium		no growth	n.a.		n.a.		neg.		neg.		neg.	
	**Microbial examination of cell-based preparation** (Ph. Eur. 2.6.27) transduction medium		no growth	n.a.		n.a.		neg.		neg.		neg.	
	**Mycoplasma** (Ph. Eur.2.6.21 and 2.6.7)		negative	neg.		neg.		neg.		neg.		neg.	
	**Endotoxin [IU/mL]** (Ph.Eur. 2.6.14)		≤ 1 IU/mL	< 0.8 IU/mL		< 0.8 IU/mL		< 0.8 IU/mL		< 0.8 IU/mL		< 0.8 IU/mL	
	**Vitality MNC [%]**		≥ 70%	98.8%		92.9%		n.a.		96.5%		98.0%	
	**Visual control**		Bag intact, no aggregates	passed		passed		passed		passed		passed	
Drug Product	**Number of cryopreserved bags**		determined, declared	n.a.		n.a.		2 × Freezing Bag 50		2 × Freezing Bag 50		4 × Freezing Bag 50, 1 × Freezing Bag 500	
	**Volume per bag [mL]**		determined, declared	n.a.		n.a.		13		84		10 ml/ Freezing Bag 50, 65 ml/ Freezing Bag 500	
	**WBC total [×10** ^**6**^ **]**		determined, declared	117.5 × 10^6^		76.7 × 10^6^		577.3 × 10^6^		452.9 × 10^6^		167.1 × 10^6^	
	**CD34 + total [×10** ^**6**^ **]**		determined, declared	101.0 × 10^6^		58.4 × 10^6^		469.6 × 10^6^		400.7 × 10^6^		160.6 × 10^6^	
	**CD34 + dose per bag [×10** ^**6**^ **/kg BW]**		determined, declared	n.a.		n.a.		n.a.		n.a.		n.a.	
	**Vitality MNC [%]** (post-thaw)		≥ 70%	91.4%		94.4%		98.6%		96.1%		98.3%	
	**Microbial examination of cell-based preparation** (Ph. Eur. 2.6.27) post cryopreservation; after addition of cryomedia before cryopreservation		no growth	neg.		neg.		neg.		neg.		neg.	
	**Clonal growth**		colony growth determined	passed		passed		passed		passed		passed	
	**Vector copy number per cell** (uncorrected measured on combined CFU-C (d12-14))		> 0	0.5		0.5		1.1		0.7		1.1	
	**Inducibility of Brec1 after tat-stimulation** [x-times increased expression (+ tat/-tat)]		Inducibility given; ≥ 3-times	54.8 ± 8.5		50.5 ± 2.0		27.8 ± 2.1		71.3 ± 6.6		18.1 ± 1.3	
	**Transduction efficiency** [Brec1 + CFU-C from total CFU-C]		> 10%	36%		29%		43%		62%		80%	
	**Visual control**		bag intact	n.a.		n.a.		passed		passed		passed	
	**Labeling**		label intact and legible	n.a.		n.a.		passed		passed		passed	
For information only	**Corrected VCN** [vector copies per cell × transduction efficiency]		> 0; FIO	1.4		1.7		2.3		1.1		1.4	
	**Mature WBCs** prior to cryopreservation	Monocytes	determined, declared; FIO	0.27%	total mature WBCs: 0.78%	0.16%	total mature WBCs: 0.73%	n.a.		0.11%	total mature WBCs: 0.55%	0.34%	total mature WBCs: 0.84%
		B-cells		0.44%		0.30%		n.a.		0.33%		0.07%	
		T-cells		0.01%		0.04%		n.a.		0.00%		0.02%	
		NK cells		0.01%		0.12%		n.a.		0.11%		0.02%	
		Eosinophils		0.01%		0.00%		n.a.		0.00%		0.01%	
		Neutrophils		0.04%		0.10%		n.a.		0.00%		0.37%	
	**Mature WBCs** post-thaw	Monocytes	determined, declared; FIO	0.56%	total mature WBCs: 1.32%	0.15%	total mature WBCs: 0.75%	0.11%	total mature WBCs: 1.00%	0.08%	total mature WBCs: 0.65%	0.17%	total mature WBCs: 0.42%
		B-cells		0.23%		0.28%		0.19%		0.28%		0.13%	
		T-cells		0.00%		0.00%		0.00%		0.03%		0.04%	
		NK cells		0.50%		0.13%		0.43%		0.23%		0.07%	
		Eosinophils		0.00%		0.00%		0.09%		0.03%		0.00%	
		Neutrophils		0.03%		0.20%		0.19%		0.03%		0.01%	

All products fulfilled all release criteria. Corrected VCN and contamination of mature cells were determined “for information only”.

## Discussion

We are presenting a partly automated manufacturing process for Brec1-engineered stem/progenitor cells leveraging the established CD34 + HSC enrichment protocol on CliniMACS Pro for CD34 + cell enrichment^33^, the novel HSC Engineering process protocol on CliniMACS Prodigy for transduction, previously only presented as abstracts and not under full cGMP conditions^34–36^, and subsequent cryopreservation using established conventional protocols for cryopreservation of stem/progenitor cells^37,38^. We demonstrate processual robustness with respect to manufacturing and quality control testing, with successful execution in five successive campaigns with cells from five different donors.

Validation work was performed with cells from G-CSF-mobilized healthy “super mobilizer” donors undergoing recipient-directed apheresis where vastly more HSPCs could be collected than were needed for the designated recipients. Apheresis material thus tended to have above-average HSPC frequencies but represented only partial apheresis products with rather lower and highly variable total HSPC doses. Later in the trial, cells from complete aphereses from otherwise healthy PLWH will be included where we may expect the entire range of mobilization responses to be represented. The maximum capacity of Prodigy is 500 × 10^6^ CD34 + cells, which we will always seek to meet, so that, accounting for the less than perfect recovery of CD34 + cell enrichment on CliniMACS Pro, a harvest dose of no less than 700–800 × 10^6^ CD34 + cells will need to be targeted during apheresis. In order to achieve this in all cases, patients may need to receive a combinatorial mobilization regime of 5 days of G-CSF plus Plerixafor in the evening preceding the apheresis. It is not anticipated that the different mobilization regimes – G-CSF in our validation, G-CSF+Plerixafor in a future trial – will affect manufacturing outcomes, as according to Lidonnici et al.^39^, mobilized HSPCs were mostly similar, irrespective of the mobilizing agent, and sufficient evidence has been provided of successful transduction of Plerixafor-mobilized stem/progenitor cells^40,41^. Also, doses of 700–800 × 10^6^ CD34 + cells were not available for any of the validation runs reported here. We thus acknowledge that in dose and quality the raw material used for validation does not perfectly represent the patient material which will later be used to manufacture IMP. We do not think, however, that these differences diminish the value of the work presented here for a future clinical trial or the cell therapy community as a whole, and the validation package was accepted by the pharmaceutical regulatory agency as sufficient to support a GMP manufacturing license for Brec1-transduced autologous HSPCs to support clinical trials.

Very short process times do not allow for meaningful decay of lentiviral particles; at the same time a high multiplicity of infection is used, high cell doses and hence, high absolute viral particle doses are required, and process-associated washing steps do not provide very high depletion of lentiviral particles. As a consequence, a finished product will contain residual lentiviral particles in the order of magnitude of 10^5^. This is equally true for other stem cell gene therapy products, which use similar MOIs and similarly short production times, and this has been found acceptable by pharmaceutical and gene technology regulatory agencies. Our target population, however, carries HIV, so that the potential risk of recombination between gene therapy vector and wild-type virus must be considered differently. A specific risk assessment was therefore performed the upshot of which is that due to the construction of the expression cassette the risk of recombination is minimal, the risks to the patient if recombination were to occur equally minimal, and hence, the risk: benefit ratio strongly in favor of the procedure. Nevertheless, monitoring for recombination events should likely be part of the follow-up of the initial few patients.

The decision to test CFU-C for certain release-determining quality attributes merits brief discussion. As mentioned above, significant quantities of free vector remain in the product at the time of harvest and cryopreservation, when most release criteria are determined. Precise quantification of vector integration for transduction efficiency and vector copy number, even if a PCR method could distinguish unintegrated and integrated vector, is not possible shortly after transduction due to the excess of pseudotransduction products (i.e. reversely transcribed, but not integrated proviral DNA). More importantly, however, we argue that these analyses in bulk freshly transduced CD34 + cells are less meaningful since the majority of CD34 + cells are mature beyond the colony-forming stage and incapable of providing engraftment. Transduction efficiency in these later progenitor cells may not be very representative of that of long-term engrafting cells.

Therefore, we elected to grow aliquots of drug product in semi-solid culture media and pick colonies for assessment of clonogenicity as well as of transduction efficiency on colony level. Determination of VCN and assessment of inducibility of the transgene by measuring Brec1 mRNA after electroporation of Tat into colony-derived, partially differentiated cells were also performed in semi-solid culture media, but in contrast to the first approach about 100 times more CD34 + cells were seeded into several 6-well plates to grow sufficient amounts of progenies for the analyses and to fit statistical expectations. The densely overgrown progeny cells were washed out from the methyl-cellulose, pooled and analysed in batch with respect to VCN and transgene inducibility (potency assay).

These analyses were performed to address the critical pharmacological specifications for purity, safety and functionality. It is acknowledged, however, that even many cells which retain the ability to grow out to colonies over two weeks are no longer endowed with engraftment potential so that even assessment at the CFU-C stage, as proposed by us, may overestimate the ultimate degree of chimerism which will establish in the patient.

CD4 T-cells cleared of integrated HIV and CD4 T-cells derived from Brec1 transduced CD34 + cells may remain susceptible to de novo HIV infection. However, this limitation does not negate the therapeutic potential of Brec1 as it will reduce the burden of replication-competent provirus and feeding of the reservoir. The planned First-in-Human-clinical trial needs to assess the potential of curing HIV.

In summary, we have developed a GMP process for manufacturing and quality control of an autologous stem cell product transduced with a designer recombinase, Brec1, for the potentially curative treatment of HIV. We present here for the first time fully clinical-scale GMP protocols for lentiviral stem cell gene therapy manufacturing and quality control on the automatic platform CliniMACS Prodigy. As we are showing, the technology is highly effective in that it reproducibly achieves satisfactory transduction rates and CD34 + cell recovery with high viability. We also propose a battery of quality control tests and specifications for our product which may provide leverage for other, similar stem cell gene therapy products. Adaptation to other gene therapy vector cargo than Brec1 should not be challenging.

## Methods

### Cells

For validation purposes, surplus cells from pseudonymized healthy mobilized donors donating for allogeneic recipients were used with their written informed consent. The experimental protocols were approved by Ethics Committee of Goethe University Faculty of Medicine, Frankfurt, Germany (vote 2021 − 111). All procedures were conducted in accordance with the Declaration of Helsinki.

### Vector

**Fig. 3:**
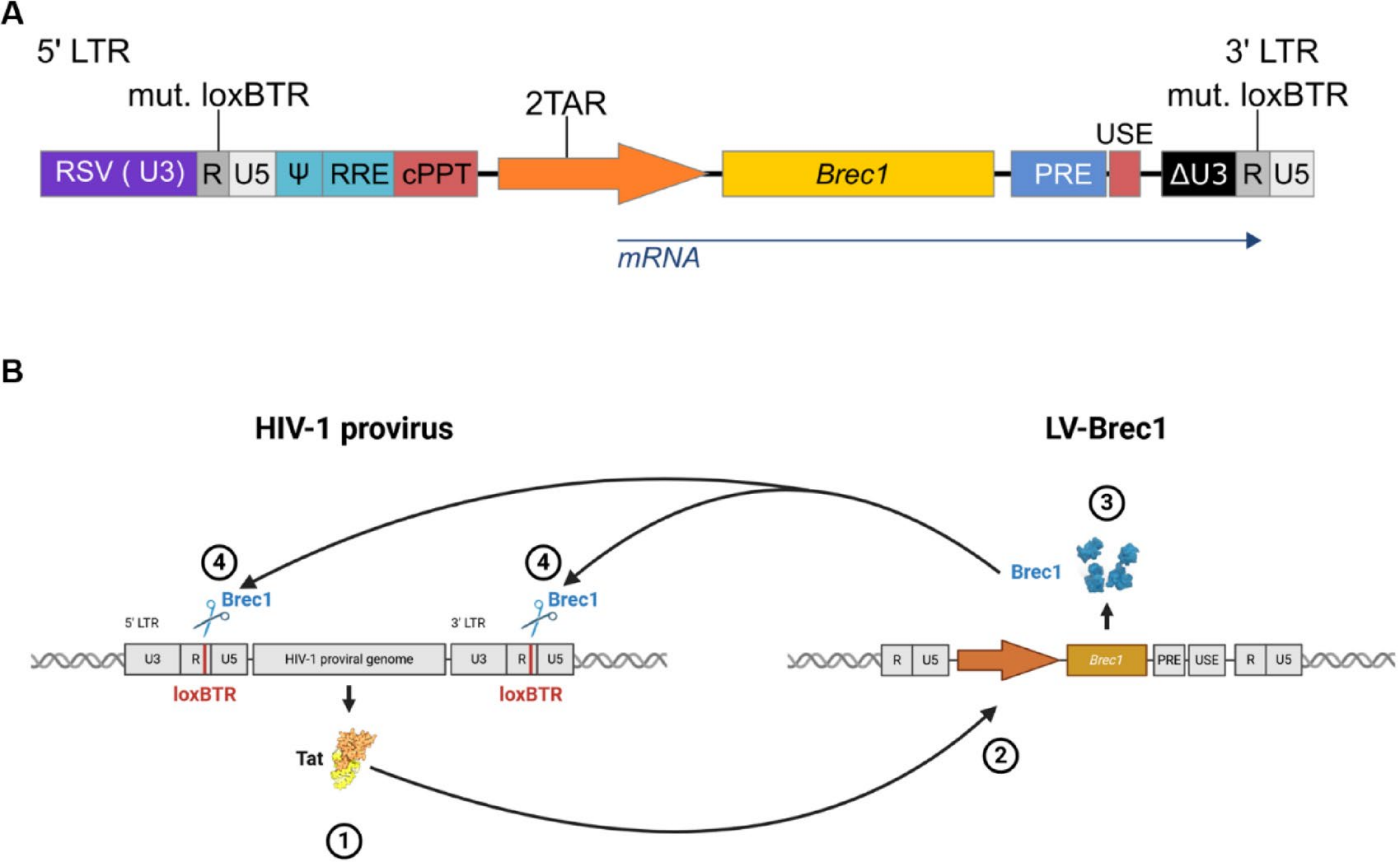
Mechanism of action of the vector cassette in HIV-infected cells. **(A)** The HIV-derived lentiviral vector backbone coding the Brec1-coding sequence contains self-inactivating (SIN) long terminal repeats (LTR: ΔU3, R, U5), a Rev response element (RRE), central polypurine tract (cPPT), posttranscriptional regulatory element derived from woodchuck hepatitis virus (PRE), SV40 upstream polyadenylation enhancer elements (USE). The transgene is placed under the control of an engineered Brec1-resistant HIV-1 L derived promoter with a tandem transactivation response element repeat (2TAR). **(B)** After administration of the ATMP (CD34 + cells transduced with LV-Brec1) the expression of Tat (1) from HIV-1 infected LV-Brec1 transduced progeny cells will result in transcription (2) and expression (3) of Brec1. Brec1 recognizes the target sequence and will excise the HIV-1 proviral genome by site specific recombination (4). This removes HIV-1 from the infected cell.

The SIN-vector containing the Brec1 (Fig. 3A) expression cassette under the control of an engineered Brec1-resistant HIV-1 L derived promoter with a tandem transactivation response element repeat (2TAR) was previously reported^25,32^. Briefly, TAR is a sequence element present in the HIV-1 genome which is recognized and bound by the transcriptional activator HIV-1 Tat. Tat expression is thus required for Brec1 transcriptional activation so that it is conditionally expressed only in HIV-1 infected cells. The mode of action of the CD34 + HSCs transduced with the SIN vector LV-Brec is demonstrated schematically in Fig. 3B. When progeny cells of LV-Brec1 transduced HSCs are infected with HIV-1, expression of Tat results in transcriptional activation of LV-Brec1 and subsequent Brec1 expression. Brec1 recognizes its target sequence in the R-region of HIV-1 L and excises the HIV-1 proviral genome by site-specific recombination, thereby removing HIV-1 from the infected cell. The loxBTR sequence of LV-Brec1 carries a targeted mutation preventing recombination of its own integrated sequence^31^.

Clinical-grade VSVG-pseudotyped lentiviral particles containing this vector cassette were manufactured under GMP-compatible protocols by a commercial vector manufacturer (Miltenyi Biotech, Bergisch-Gladbach, Germany). Vector was supplied frozen in HSA-saline in EVA-bags in controlled-rate deep freezers and stored at -80 °C until just prior to use. The relatively low-titer vector necessitated aggregation of the contents of several (up to twenty) bags in an A-in-B environment before sterile-welding of the vector bag to a Tubing Set 520 of CliniMACS Prodigy.

### Reagents and consumables for manufacturing

Unless otherwise mentioned, all reagents and consumables were commercial GMP-grade products that were used within the manufacturer’s specifications. CD34 selection reagent, buffers and tubing set were from Miltenyi Biotech, pharmaceutical-grade human serum albumin from CSL Behring (Marburg, Germany), CliniMACS Prodigy Tubing Set 520, HSC Brew media and supplements, as well as washing and elution buffers were from Miltenyi Biotech, as were CliniMACS cryopreservation bags. The transduction-enhancer LentiBOOST was sourced from Sirion Biotech (Gräfelfing, Germany), pharmaceutical-grade DMSO from WAK Chemie (Steinbach, Germany). CliniMACS Pro and CliniMACS Prodigy are from Miltenyi Biotech, BioFreeze 45 from Consarctic (Westerngrund, Germany).

### Manufacturing process

**Fig. 4:**
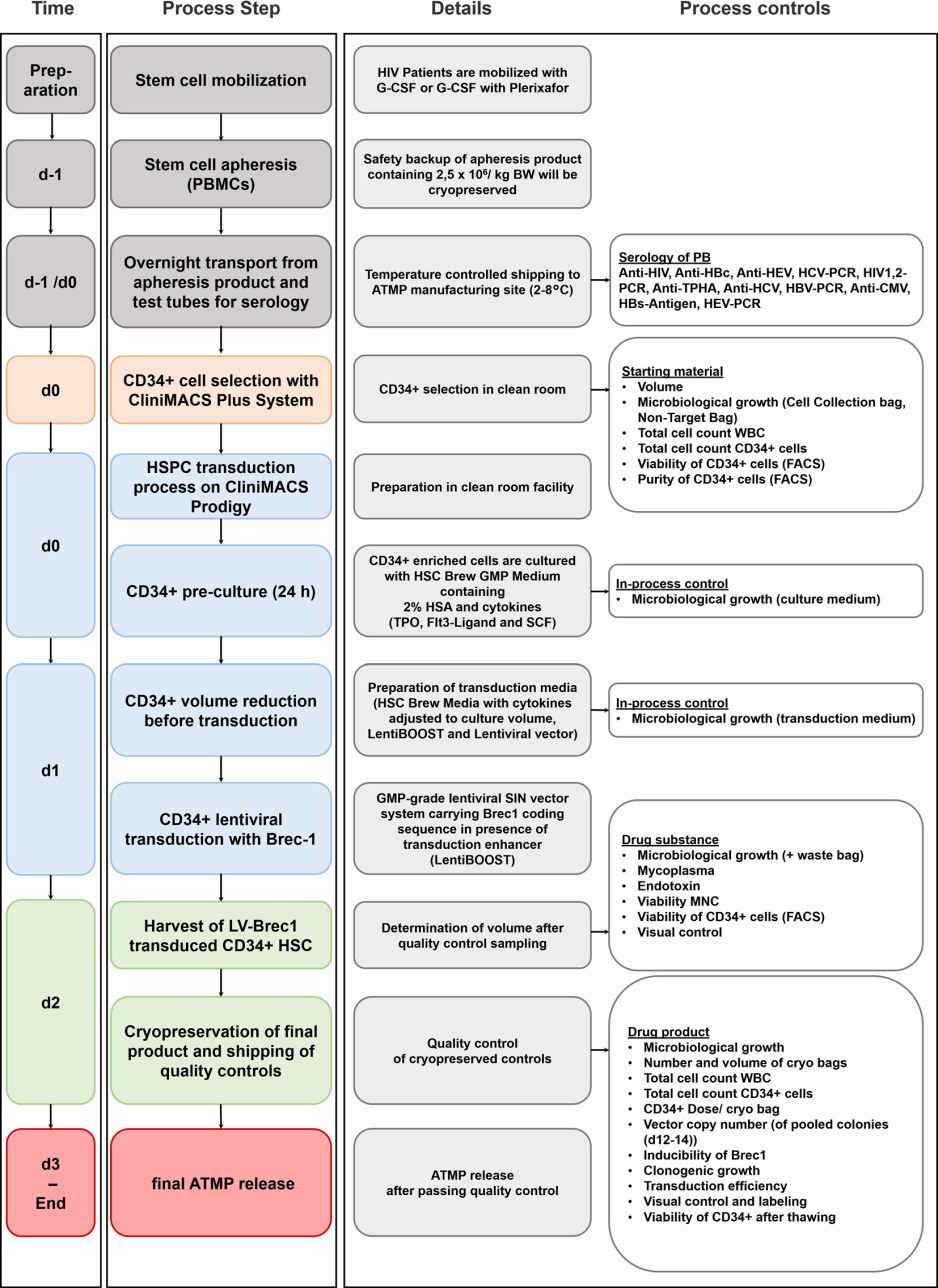
Workflow diagram of the ATMP manufacturing procedure with timeline and quality controls.

G-CSF-mobilized apheresis product was used as starting material (see above). After a soft-spin for platelet reduction, CD34 + cell enrichment using, depending on cell number, LS or TS tubing systems, ensued, as previously described^42,43^. CD34 + cells were harvested in CliniMACS PBS/EDTA + 0.5% HSA and connected to the CliniMACS Prodigy at valve 8 of tubing set 520 within the hematopoietic stem cell engineering (HSCE) process. A maximum of 500 × 10^6^ CD34 + cells were loaded on the system, resulting in a CD34 + cell concentration of no higher than 2.5 × 10^5^/mL with 200 mL as maximal volume within the CentriCult Unit. The cells were cultivated in HSC Brew medium supplemented with 300 U/mL SCF, 270 U/mL FLT3-L and 1600 U/mL TPO. 24 h after inoculation the CliniMACS Prodigy automatically reduced the culture volume down to 10 mL. Transduction was performed with room temperature equilibrated Brec-1 LV at an MOI of 120–140 and 2% LentiBOOST. Culture volume was re-adjusted to initial culture volume with cytokine-replete (see above for concentrations) HSC Brew. 16 h later, cells were twice washed and harvested in 0.9% NaCl + 0.5% HSA into the target cell bag 1 of the CliniMACS Prodigy TS 520 tubing system. Drug substance volume was gauged by net weight; an equal volume of double-strength cryopreservation medium was added aseptically in a closed system, to achieve a final concentration of HSA of 3% w/v and DMSO 7.5% v/v in the drug product. Aliquots for pre-freeze quality control tests on the drug product as well as for at least six retain samples were collected, then the cell product was frozen in a controlled-rate freezer with heat dissipation at the eutectic point. A typical patient dose will consist of one bag, containing the entirety of the cells harvested from one manufacturing run, up to 500 × 10^6^ cells minus QC and retain samples to the targeted patient dose will thus be 3–7 × 10^6^ CD34 + cells, a variable fraction thereof Brec1 transduced, per kg body weight. The individual steps of the manufacturing procedure including quality controls are summarized as a flow chart in Fig. 4.

### Quality control

The sampling algorithm is depicted in the manufacturing flow chart (Fig. 4). Microbial safety was tested acc. to Ph.Eur. 2.6.27, using the BacT/Alert (BioMerieux, Marcy-l’Étoile, France) system, with incubation at 30 °C for the aerobic and 36 °C for the anaerobic bottle. Endotoxin was measured acc. to Ph.Eur. 2.6.14 with a colorimetric Limulus amebocyte lysate assay (Endosafe, Charles River, Wilmington, USA). Mycoplasma was tested using a NAT system in accordance with Ph.Eur. 2.6.7 and Ph.Eur. 2.6.21 (Sartorius, Göttingen, Germany). CD34 + cell enumeration including a vital dye was performed acc. to Ph.Eur. 2.7.24 by flow cytometry with the CE-marked SCE kit (BD Biosciences, Heidelberg, Germany)^33^. Cellular impurities were characterized and quantified with an established multi-color flow cytometry panel as previously reported^44,45^. Novel assays were established for assessment of the more specific pharmacological properties of the Brec1 modified HSC product. Thus transduction efficiency was assessed by culturing small aliquots (few hundred transduced cells) of drug product in semi-solid cytokine-replete methylcellulose medium for 12–14 days (Methocult3434, Stem Cell Technology, Vancouver, BC). Individual colonies of differentiated myeloid cells grown in the semi-solid medium from stem/progenitor cells were picked into wells of four 96-well plates and air-dried. From each colony DNA was extracted individually, then subjected to real-time PCR using a PCR recognizing Brec1 (FAM) and RnaseP (gDNA control gene, VIC). Transduction efficiency was calculated as the fraction of positive Brec1 PCR reactions (transduced colony) among control gene positive PCR reactions.

For assessment of the vector copy number, 6 × 10^4^ CD34 + cells were seeded into semi-solid methylcellulose and plated on several 6-well plates. 12 days after seeding, densely overgrown cells were harvested by washing the cells out of the methylcellulose, and from 5 × 10^5^ cells genomic DNA was isolated and ddPCR was performed to assess the relative number of Brec1 positive (transgene) and RPP30 positive (gDNA control) droplets. Functionality of the transgene was assessed by electroporation of 6 aliquots of 2 × 10^6^ of the harvested cells with recombinant Tat protein, 6 more aliquots of 2 × 10^6^ cells were used as negative controls. After 40–48 h in culture, total RNA was isolated, transcribed into cDNA and ddPCR was performed to assess the relative number of Brec1 positive and RPL13A positive droplets. Targeted specifications together with the results from the individual validation runs for all quality parameters are shown in Table 1.

### Stability program

For one of the manufacturing campaigns, the drug product was frozen not in one, but in 44 individual small vials and 5 bags, to facilitate thawing at the earliest possible time (time point 0), as well as after 1, 3, 6 and 12 months, to produce stability data which might support a shelf life of up to 12 months, even though we anticipate re-infusing the drug product very soon after completion of the quality assays, i.e. within an estimated 2 months after freezing. The test matrix and specification of post-thaw analyses is depicted in Table 2; the same battery of assays is used as for quality controls of clinical batches for QP release, but not all assays at all time points. At the time of this writing, data are available for the first three time points (up to three months).

**Table 2:** Pre-defined specifications for the stability analysis of the frozen drug product.

	Test parameter		Specification for drug product	Specification for stability study	1 month	3 month	6 month	12 month
Drug Product	**Microbial Examination of cell-based Preparation** (Ph. Eur. 2.6.27) post cryopreservation; after addition of cryomedia before cryopreservation		no growth	no growth	-	✓	-	✓
	**Mycoplasma** (Ph. Eur.2.6.21 and 2.6.7)		negative	negative	-	✓	-	✓
	**Endotoxin [IU/mL]** (Ph.Eur. 2.6.14)		≤ 1 IU/mL	≤ 1 IU/mL	-	✓	-	✓
	**Vitality MNC [%]** (post-thaw)		≥ 70%	reference value ± 10%	✓	✓	✓	✓
	**WBC total [×10** ^**6**^ **]**		determined, declared	determined, declared	✓	✓	✓	✓
	**CD34 + total [×10** ^**6**^ **]**		determined, declared	determined, declared	✓	✓	✓	✓
	**Clonal growth**		colony growth determined	colony growth determined	✓	✓	✓	✓
	**Vector copy number per cell** (uncorrected measured on combined CFU-C (d12-14))		> 0	reference value ± 3	✓	✓	✓	✓
	**Inducibility of Brec1 after tat-Stimulation** [x-times increased expression (+ tat/-tat)]		Inducibility given; ≥ 3-times	Inducibility given; ≥ 3-times	✓	✓	✓	✓
	**Transduction efficiency** [Brec1 + CFU-C from total CFU-C]		> 10%	reference value ± 10%	✓	✓	✓	✓
	**Labeling**		label intact and legible	label intact and legible	-	✓	-	✓
For information only	**corrected VCN** [vector copies per cell × transduction efficiency]		> 0; FIO	determined, declared	✓	✓	✓	✓
	**mature WBCs** prior to cryopreservation	Monocytes	determined, declared; FIO	determined, declared	-	✓	-	✓
		B-cells						
		T-cells						
		NK cells						
		Eosinophils						
		Neutrophils						

(- not tested, ✓ to be tested at the corresponding time points)

### Aseptic process validation

**Fig. 5:**
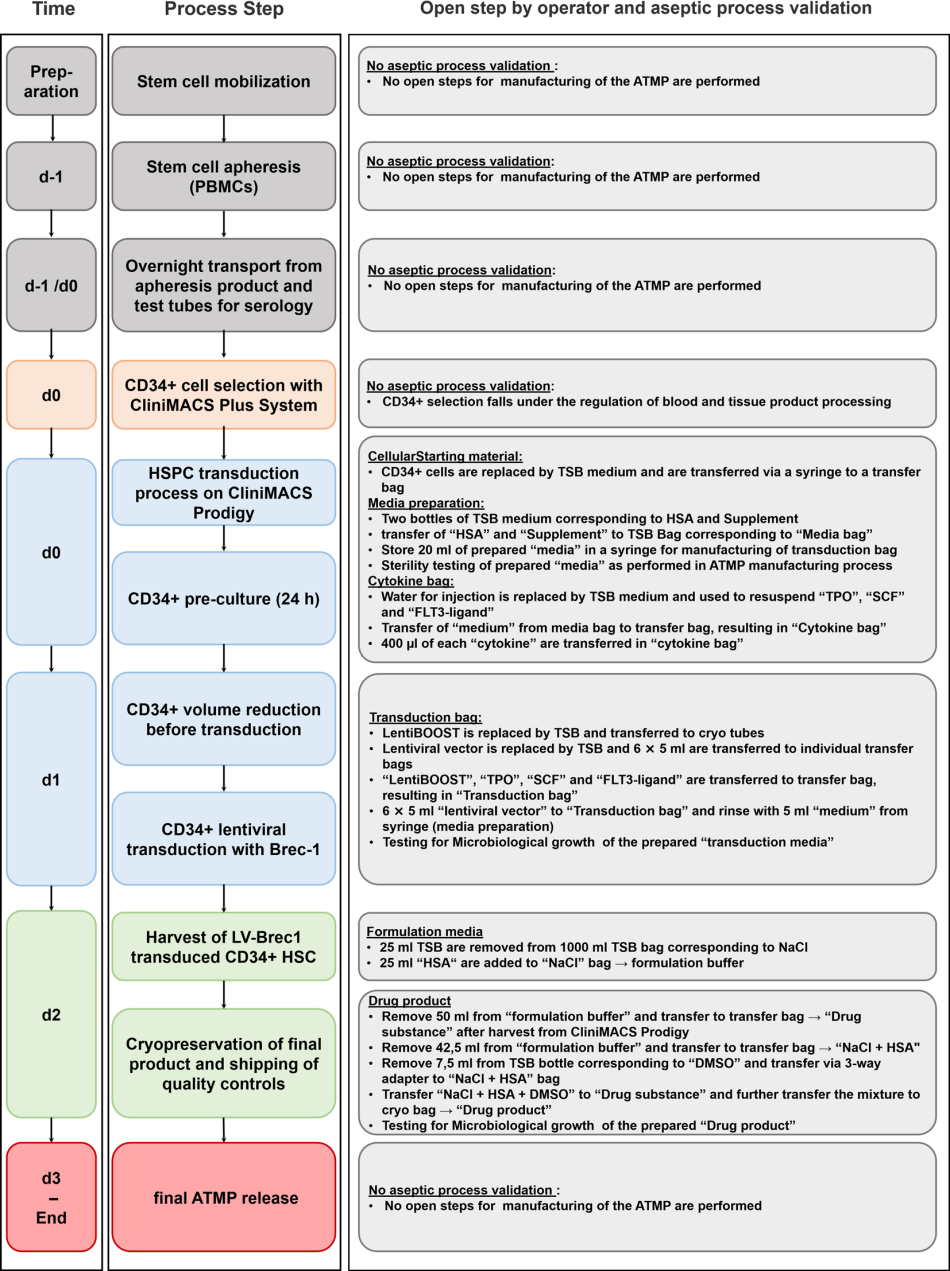
Workflow diagram of the approved aseptic process validation for Brec1-transduced HSPCs.

Despite the succession of three closed processes, aseptic process validation is expected, solely for the reason that this is an ATMP. We designed, therefore, an aseptic process validation protocol, the flow chart of which, including the process steps that are simulated by the specific activities, is given in Fig. 5.

## Data Availability

The data that support the findings of this study are available within the manuscript. Additional data related to this paper are available from the corresponding author upon reasonable request, in accordance with this journal’s Share Upon Reasonable Request data policy.
